# Updated description of *Diospyrosdussaudii* Lecomte (Ebenaceae), with lectotypification and notes on its distribution

**DOI:** 10.3897/phytokeys.184.71045

**Published:** 2021-11-04

**Authors:** Sutee Duangjai, Aroon Sinbumroong, Porntawat Chalermwong, Padungsak Suekaew, Kwanjai Khammongkol, Wittawat Kiewbang, Sukid Rueangruea, Theerawat Thananthaisong, Somran Suddee

**Affiliations:** 1 Department of Forest Biology, Faculty of Forestry, Kasetsart University, Bangkok, 10900, Thailand; 2 Protected Area Regional Office 4 (Surat Thani), Department of National Parks, Wildlife and Plant Conservation, Surat Thani, 84000, Thailand; 3 Surat Thani National Park and Protected Area Innovation Center, National Park Innovation Institute, Department of National Parks, Wildlife and Plant Conservation, Surat Thani, 84000, Thailand; 4 Phu Langka National Park, Department of National Parks, Wildlife and Plant Conservation, Nakhon Phanom, 48140, Thailand; 5 Forest Economics Bureau, Royal Forest Department, Bangkok 10900, Thailand; 6 Forest Herbarium (BKF), Department of National Parks, Wildlife and Plant Conservation, Bangkok, 10900, Thailand

**Keywords:** Dioecy, *Diospyros*, DNA sequence data

## Abstract

*Diospyrosdussaudii* is a poorly known species and previous descriptions lacked details about its female flowers and fruits. The species had not been recorded since type collections were made in 1913. As a result of our *Diospyros* research in Thailand, new specimens and data are now available for this species. In this study, we provide an updated morphological description, illustrations, lectotypification and a distribution map. The species was previously reported to be endemic to Laos; as such, the occurrences in Thailand greatly expand the distribution of the species. In addition, we analysed the phylogenetic relationships between *D.dussaudii* and other *Diospyros* species from Southeast Asia and other regions, using DNA sequence data from eight plastid regions. Our phylogenetic analyses indicate that *D.dussaudii* is closely related to *D.castanea*, *D.dasyphylla* and *D.insidiosa*. Their taxonomic affinities are discussed.

## Introduction

Although the taxonomy of the genus *Diospyros* L. in Thailand is fairly well documented, some specimens do not fit species in the known flora of Thailand (Phengklai 1981, 2005; Duangjai et al. 2018, 2020; Eiadthong 2020). This was the case for specimens collected from Phu Langka National Park in Nakhon Phanom Province, north-eastern Thailand in 2013 and from Chumphon Province, peninsular Thailand in 2014. The leaves and buds of these specimens resemble those of *D.dasyphylla* Kurz, which also occurs in Thailand. However, their fruits are globose or depressed-globose, ca. 4.0–5.0 cm in diameter and densely covered with orange hairs, similar to those of *Sandoricumkoetjape* (Burm. f.) Merr. (Meliaceae), but different from those of *D.dasyphylla*, which has glabrous fruits. When we compared these specimens with species known to occur in Indochina (Lecomte 1928, 1930), as well as specimens from the Muséum national d’Histoire naturelle (P), we observed similarities in the leaves and buds to a poorly known species from Laos, *D.dussaudii* Lecomte. However, we could not positively identify the specimens due to lack of detail on the female plants of *D.dussaudii* in the published descriptions and due to the absence of male specimens amongst our collections.

*Diospyrosdussaudii* is one of thirty Indochinese *Diospyros* species described by the French botanist Paul Henri Lecomte (1856–1934) in 1928 (Lecomte 1928). Lecomte’s description was based only on male specimens, which were collected by M. Dussaud from what is now the Lao People’s Democratic Republic (PDR) on 4 October 1913. Until recently, the species was known only from one collection with three type specimens (P00721485, P00721486 and P02141495) held at P. The species is poorly known and was previously reported to be endemic to Laos (Lecomte 1928, 1930). It was not thought to occur in Thailand (Phengklai 1981) or China (Lee et al. 1996) and is not included in a checklist of vascular plants of the Lao PDR (Newman et al. 2007a). No information was available about the female flowers and fruits, which are important in the systematics of *Diospyros* and no specific locality was mentioned in the protologue (Lecomte 1928).

In 2019, Kwanjai Khammongkol collected additional specimens of the same unknown *Diospyros* species from Tat Pho Waterfall, Phu Langka National Park, Thailand. Later, in 2020, additional populations of this species were found in peninsular Thailand during floristic surveys conducted by teams from the Protected Area Regional Office 4 (Surat Thani) and the Surat Thani National Park and Protected Area Innovation Centre. Female and male flowers were collected from Surat Thani Province and Chumphon Province, respectively. Based on examination of type specimens at P, we identified a male specimen of the unknown *Diospyros* from peninsular Thailand as *D.dussaudii*.

In late December 2020, Sukid Rueangruea and Somran Suddee found a sapling of *D.dussaudii* during a field trip with Japanese botanists on the Bolaven plateau in southern Laos, but did not collect it. However, after searching for Laotian specimens in various herbaria, another Laotian collection (Newman et al. LAO 833) of *D.dussaudii* was found in E and L (L0409075). These specimens were collected in 2005 from Khammouan and were initially identified as *Diospyros* sp.

The objectives of this study were to report the rediscovery of *D.dussaudii* in Laos and provide an updated description of the species, as well as photographs, illustrations and notes on its distribution. In addition, we typified the species name and selected a lectotype. We also determined the phylogenetic placement of the species using DNA sequence data. Finally, to facilitate the distinction of *D.dussaudii* from closely allied species, a comparison of morphological characters is presented.

## Materials and methods

### Morphological investigation, description and geographical distribution

Examination of *D.dussaudii* was based on specimens and preserved spirit collections obtained from north-eastern and peninsular Thailand. These voucher specimens, representing both male and female plants, were deposited in the Bangkok Herbarium (BK) and Bangkok Forest Herbarium (BKF). We also examined digital images of specimens held at BM, E, K, L and P (abbreviations follow Thiers 2020). We further compared these specimens with all published records of *Diospyros* species in Thailand and adjacent regions (Lecomte 1930; Bakhuizen van den Brink 1936–1955; Ng 1978, 2001; Phengklai 1981, 2005; Lee et al. 1996; Singh 2005). Material collected from north-eastern and peninsular Thailand was photographed in the field. The habit, habitat, coordinates and elevation were documented in the field. Floral morphology was studied with dissecting microscopes at the Department of Forest Biology, Faculty of Forestry, Kasetsart University. An updated description of the species was developed from digital images of type specimens from P, the protologue, digital images of Laotian specimens held at L, specimens collected from Thailand and field observations. A distribution map, based on specimens and field observations, was created with SimpleMappr (Shorthouse 2010). The conservation status of the species was evaluated with IUCN Red List Categories and Criteria (IUCN 2019).

### Phylogenetic analysis

One accession of *D.dussaudii* from north-eastern Thailand and three accessions from peninsular Thailand were compared with DNA sequences of eight plastid regions (*rbcL*, *atpB*, *matK*, *ndhF*, *trnK* intron, *trnL* intron, *trnL*-*trnF* spacer and *trnS-trnG* spacer). Total DNA was extracted from silica-dried leaf samples with a modified 2× cetrimonium bromide procedure (Doyle and Doyle 1987). The primers and polymerase chain reaction (PCR) protocol used for amplification are as described in Duangjai et al. (2009), except that we used 2× DreamTaq Green PCR Master Mix (Thermo Fisher Scientific, Waltham, MA, USA), following manufacturer’s protocols. Successfully amplified products were cleaned with FastAP Thermosensitive Alkaline Phosphatase and Exonuclease I (Thermo Fisher Scientific). The cleaned PCR products were sequenced with the same primers used in the initial amplifications. Sanger sequencing was performed at the Macrogen sequencing facility (Macrogen, Inc., Seoul, South Korea).

The DNA sequences of *D.dussaudii* were manually aligned to the dataset from Duangjai et al. (in prep.). Phylogenetic analyses were carried out with Maximum Parsimony (MP) and Bayesian Inference (BI; Rannala and Yang 1996; Yang and Rannala 1997). The MP analyses were conducted with equally weighted, unordered nucleotide substitutions (Fitch 1971) in PAUP* v.4.0b10 (Swofford 2002). The most parsimonious trees were searched heuristically with 1,000 replicates of random sequence addition; the settings included tree bisection and reconnection (TBR) swapping and MulTrees = on. TBR swapping was performed on a maximum of 200 trees (nchuck = 200) per replicate. Node support was evaluated with 1,000 bootstrap replicates of 1,000 random additions. BI was performed with MrBayes v.3.2 (Ronquist et al. 2012) on the CIPRES Science Gateway platform (Miller et al. 2010). Nucleotide substitution models were selected with the Akaike Information Criterion (AIC), implemented in MrModeltest v.2.3 (Nylander 2004). We performed two independent Markov chain Monte Carlo analyses, with four simultaneous chains of 10,000,000 generations, sampling one tree per 1,000 generations. The first 25% were discarded as burn-in and the remaining trees were used to construct a majority-rule consensus tree with Bayesian Posterior Probabilities (PPs). *Euclea* L., *Lissocarpa* Benth. and *Royena* L. (Ebenaceae) species were selected as the outgroup. Genetic distances between *D.dussaudii* and closely related species were generated with the Kimura 2-parameter model (Kimura 1980), with all gaps treated as missing (complete deletion option). DNA sequences of the eight plastid regions from four individuals of *D.dussaudii* were submitted to GenBank (accession numbers: MZ457089–MZ457112).

We compared the morphology of *D.dussaudii* with that of three species determined to be closely related on the basis of the results of the phylogenetic analyses. Data for *D.castanea*, *D.dasyphylla* and *D.insidiosa* Bakh. were obtained from previous studies (Lecomte 1930; Bakhuizen van den Brink 1936–1955; Ng 1978; Phengklai 1981; Lee et al. 1996; Singh 2005) and supplemented by our own observations.

## Results and discussion

**Figure 1.**
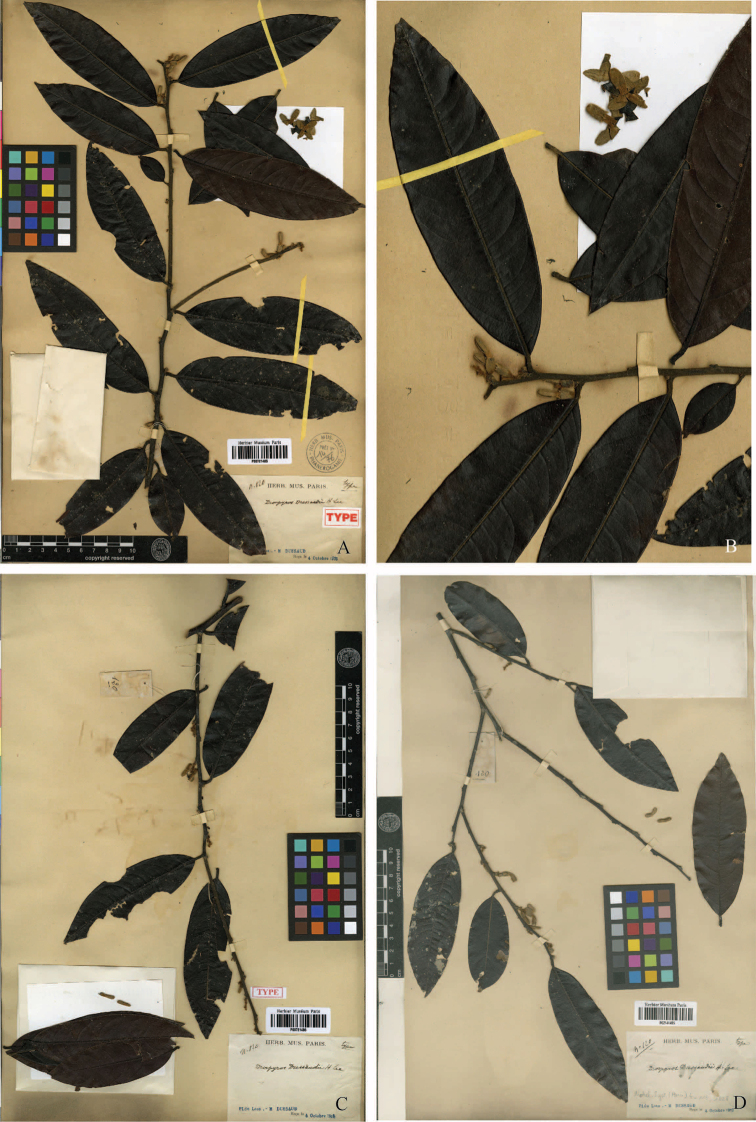
Type specimens of *D.dussaudii* (M. Dussaud 120) deposited at P. **A, B** lectotype, P00721485, **C** isolectotype, P00721486 and **D** isolectotype, P02141495.

After careful study of the protologue and type specimens (Fig. 1), we determined that our collections from peninsular Thailand matched the description and type specimens of *D.dussaudii*. *Diospyrosdussaudii* is a poorly known species and the protologue included a description of male plants only. The rediscovery of *D.dussaudii* in Laos and the recent collections from Thailand allowed us to complete the description of the species.

### Phylogenetic analysis

**Figure 2.**
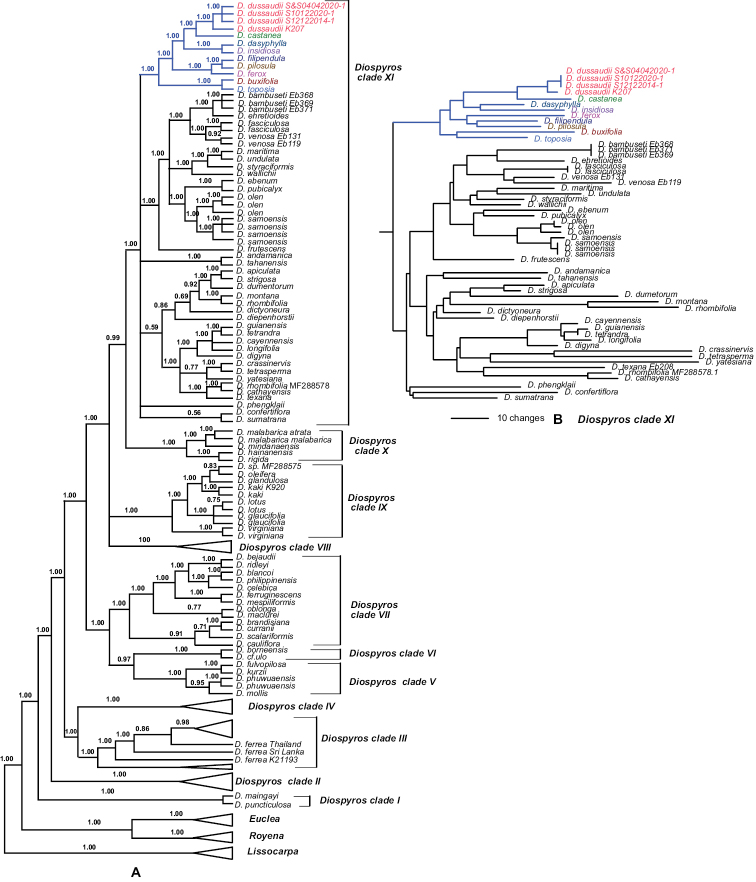
Phylogenetic trees of *Diospyros* and related genera, based on DNA sequence data from eight plastid regions **A** the 50% majority-rule tree from BI analysis. Posterior probabilities > 0.50 are shown above branches **B** phylogram from BI analysis, showing only the details of *Diospyros* clade XI. *Diospyrosdussaudii* and closely related species are indicated in coloured text.

We investigated the phylogenetic relationships of one individual of *D.dussaudii* from north-eastern Thailand and three individuals from peninsular Thailand using DNA sequence data from eight plastid regions. In addition, we clarified the phylogenetic relationships between *D.dussaudii* and other *Diospyros* species. When sequences of four individuals of *D.dussaudii* were included in the data matrix, the concatenated alignment of the 186-terminal dataset consisted of 8,293 characters, amongst which 1,991 sites were variable and 1,150 were MP-informative. MP analysis yielded 23,000 equally parsimonious trees with 3,484 steps (consistency index = 0.66; retention index = 0.84). The results of the MP and BI analyses were generally congruent; therefore, we present only the latter (Fig. 2). Phylogenetic analyses of eight plastid regions indicate with strong support (PP 1.0) that *Diospyros* and three other genera, *Euclea*, *Lissocarpus* and *Royena*, are monophyletic. The overall phylogenetic relationships of these four genera and of the clades recovered within *Diospyros* are congruent with previous reports (Duangjai et al. 2009, 2018, 2020). We identified 11 major clades of *Diospyros*, eight of which (I, III, V, VI, VII, IX, X and XI) include Asian species. The results of our phylogenetic analyses unambiguously place the four *D.dussaudii* individuals within clade XI (Fig. 2) with 43 other species from Asia, the Americas, New Caledonia and the Pacific Islands. Although relationships within the clade are not fully resolved, the four individuals of *D.dussaudii* group together with high support (PP = 1.00) and have a well-supported sister relationship with *D.castanea* (PP = 1.00) and this clade is sister to a clade of *D.dasyphylla* and *D.insidiosa*. The genetic distance between *D.dussaudii* and *D.castanea*, *D.dasyphylla* and *D.insidiosa*, based on data from eight plastid regions, ranges from 0.0041 to 0.0080, whereas intraspecific distances amongst the four individuals of *D.dussaudii* range from 0 to 0.0009 (Table 2). Pairwise similarity for *D.dussaudii* is highest with *D.castanea* (99.58%), followed by *D.dasyphylla* (99.36%) and *D.insidiosa* (99.20%). There are 13 polymorphic characters out of 8,293 that differ within the four individuals of *D.dussaudii*. However, the four individuals have 27 characters in common that differ from the sister species *D.castanea*.

Other species in the same subclade as *D.dussaudii* are *D.buxifolia* Thouars, *D.ferox* Bakh., *D.filipendula* Pierre ex Lecomte, *D.pilosula* Wall. ex Hiern and *D.toposia* Buch.-Ham. The nine species in the subclade are all distributed in South or Southeast Asia (Lecomte 1930; Bakhuizen van den Brink 1936–1955; Ng 1978; Phengklai 1981).

Amongst the species in the subclade, the fruits of *D.dussaudii* are similar in size to those of *D.castanea*, *D.dasyphylla* and *D.insidiosa*. They are globose or depressed-globose and ca. 4.0–5.0 cm in diameter, whereas *D.buxifolia*, *D.ferox*, *D.filipendula* and *D.pilosula* have fruits that are less than 2 cm in diameter. The differences between *D.dussaudii* and *D.castanea*, *D.dasyphylla* and *D.insidiosa* are shown in Table 1. When vegetative, *D.dussaudii* may be confused with *D.dasyphylla*; however, the two species can be distinguished by leaf texture and the shape of the leaf base. *Diospyrosdussaudii* has thicker leaves than *D.dasyphylla*. The leaf base of *D.dussaudii* is attenuate or cuneate, whereas that of *D.dasyphylla* is obtuse or cordate. In addition, the fruits of *D.dussaudii* are hairy, with a thin mesocarp (ca. 3.0–4.0 mm thick), whereas *D.dasyphylla* has shiny glabrous fruits with a thicker mesocarp (ca. 10.0 mm). The outer bark of *D.dussaudii* is smooth and has sparse lenticels, whereas *D.dasyphylla* has scaly bark without lenticels. When one compares leaves of *D.dussaudii* with those of *D.castanea* and *D.insidiosa*, the leaves of the latter two species are glabrous on both surfaces. The fruiting calyces of *D.dussaudii*, *D.dasyphylla* and *D.insidiosa* are similar and slightly enlarged compared to the flowering stage, with four spreading, rounded lobes, ca. 4.0 × 5.0 mm, that are divided to the base. The calyces of *D.castanea*, by contrast, are discoid and spreading, ca. 14 mm in diameter and entire or sometimes split into three lobes.

**Table 1.** Morphological differences between *D.dussaudii* and closely related species.

Character	*D.dussaudii*	*D.castanea*	*D.dasyphylla*	*D.insidiosa*
Bark	Outer bark grey, smooth with sparse lenticels, inner bark reddish-brown or blackish-brown, thick	Outer bark grey, cracked or scaly, inner bark brown, thick	Outer bark brownish-grey, scaly without lenticels, inner bark reddish-brown, thin	Outer bark greenish-black, smooth, inner bark blackish-brown, thick
Bifarious scales covering bud	Present	Present	Present	Absent
Leaves	Oblong or elliptic-oblong, 12.0–16.2 × 3.8–5.0 cm, subcoriaceous, glabrous adaxially, glabrous to tomentose abaxially, base attenuate or cuneate, apex acute to acuminate	Ovate, oval or elliptic, 6.0–13.0 × 2.5–8.0 cm, subcoriaceous or coriaceous, glabrous on both surfaces, base rounded, truncate or subcordate, apex acute to acuminate	Elliptic, ovate-oblong, oblong or obovate, 7.0–20.0 × 3.5–8.0 cm, chartaceous, glabrous adaxially, hispid or tomentose abaxially, base obtuse or cordate, apex acuminate to caudate-acuminate	Ovate or oblong, 5.0–17.0 × 2.0–7.0 cm, chartaceous to subcoriaceous, glabrous on both surfaces, base acute or obtuse, apex acute to acuminate
Merosity	4-merous	3-merous	4-merous	4–5-merous
Staminodes	8	Absent	Unknown	4–8
Fruit shape, covering and size	Globose or depressed-globose, covered with dense orange hairs, 3.8–5.0 × 4.0–5.0 cm	Globose or ellipsoid, glabrous or pubescent near base, 2.0–5.0 × 2.0–5.0 cm	Globose or depressed-globose, glabrous and shiny, 4.0–5.0 × 5.0–6.0 cm	Globose or depressed-globose, glabrous and shiny, 4.0–5.0 × 5.0–7.0 cm
Fruiting calyx	Lobes 4, divided to the base, ovate-oblong, spreading, ca. 4.0 × 5.0 mm	Discoid and spreading, ca. 14.0 mm in diameter, entire or sometimes split into 3 lobes	Lobes 4, divided to the base, ovate-oblong, spreading, ca. 4.0 × 5.0 mm	Lobes 4 or 5, divided to the base, ovate-oblong, spreading, ca. 4.0 × 5.0 mm
Mesocarp of mature fruits	Cream with brown dots, 3.0–4.0 mm thick	Cream with brown dots, 3.0–4.0 mm thick	Cream with brown dots, ca. 10.0 mm thick	Yellow, ca. 10.0 mm thick
Seeds	3–8 seeds per fruit, ellipsoid to planoconvex, endosperm slightly ruminated when mature	1–4 seeds per fruit, subglobose or ellipsoid, endosperm ruminated	6–8 seeds per fruit, ellipsoid to planoconvex, endosperm smooth	6–8 seeds per fruit, ellipsoid to planoconvex, endosperm smooth

**Table 2.** Kimura 2-parameter genetic distance amongst four individuals of *D.dussaudii* and closely related species.

	1	2	3	4	5	6
1. *D.dussaudii* S12122014-1						
2. *D.dussaudii* S&S04042020-1	0.0000					
3. *D.dussaudii* S10122020-1	0.0000	0.0000				
4. *D.dussaudii* K207	0.0009	0.0009	0.0009			
5. *D.castanea*	0.0042	0.0042	0.0042	0.0041		
6. *D.dasyphylla*	0.0064	0.0064	0.0064	0.0064	0.0063	
7. *D.insidiosa*	0.0080	0.0080	0.0080	0.0079	0.0080	0.0049

### Taxonomy

#### Diospyros dussaudii

Taxon classificationPlantaeEricalesEbenaceae

Lecomte, Notul. Syst. (Paris) 4: 113. 1928; in Fl. Indo-Chine 3: 954. 1930.

2FE0C846-309B-522E-ADA7-0B7576548638

##### Type.

**Figure 3.**
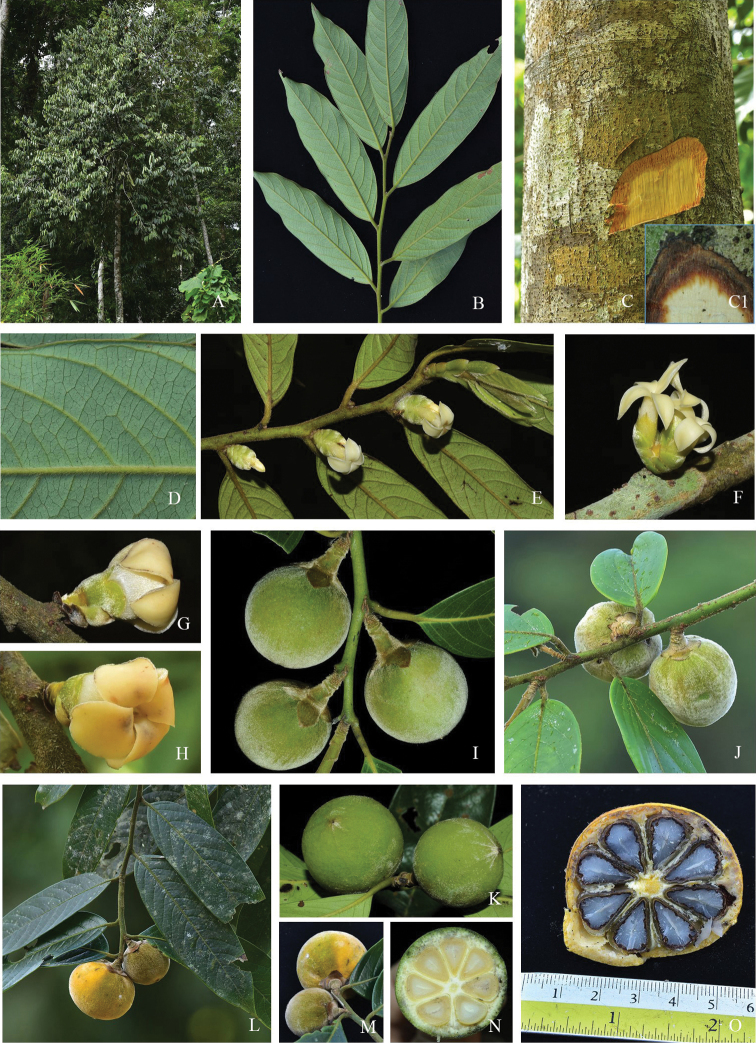
*Diospyrosdussaudii***A** habit **B** branch and leaves **C** trunk and bark **C1** slash of bark **D** leaf venation, abaxial view **E, F** male inflorescences **G, H** female flower **I–K** immature fruits **L, M** mature fruits **N, O** fruit in cross section showing immature seeds (**N**) and mature seeds (**O**). Photographs by Aroon Sinbumroong (**A–H, J, L–M** and **O**) and Sukid Rueangruea (**I, K** and **N**).

**Figure 4.**
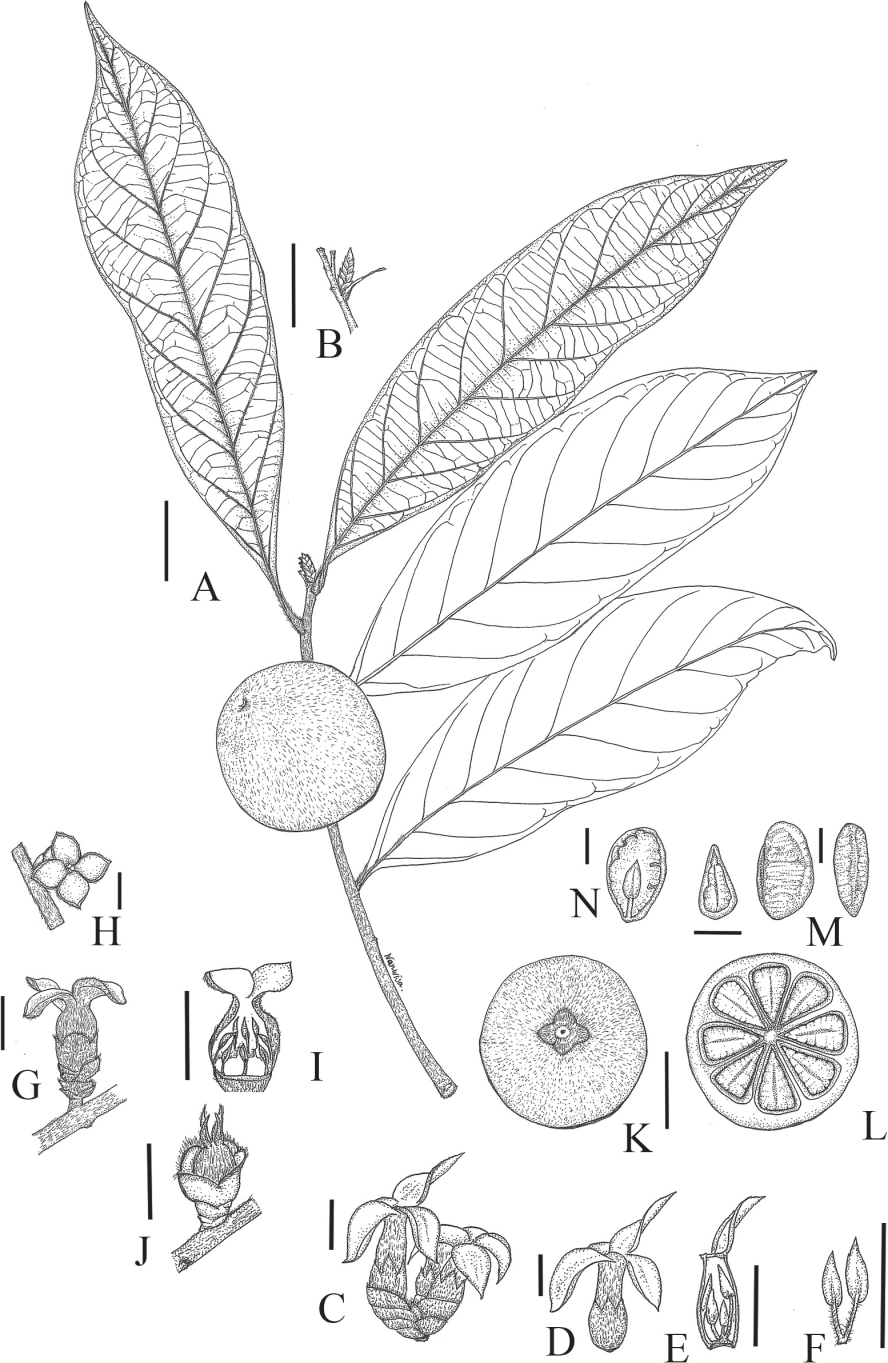
*Diospyrosdussaudii***A** fruiting branchlet **B** axillary bud **C** male inflorescence **D** male flower **E** opened corolla of male flower, showing stamens **F** stamens **G** female flower, side view **H** female flower, top view **I** opened corolla of female flower, showing eight staminodes attached at the base of corolla tube **J** ovary and stigma of female flower **K** fruit with four calyx **L** cross section of 8-seeded fruit **M** seeds, top view and side view and **N** the longitudinal section of seed, showing embryo. Drawn by W. Bhuchaisri from *Sinbumroong & Suekaew 04042020-1* (**A, B** and **K–N**), *Sinbumroong 12092020-1* (**C–F**) and *Sinbumroong 08052020-1* (**G–J**).Scale bars: 1 cm (**G–J, M, N**); 2 cm (**A, B, K, L**); 5 mm (**D–F**); 8 mm (**C**).

**Figure 5.**
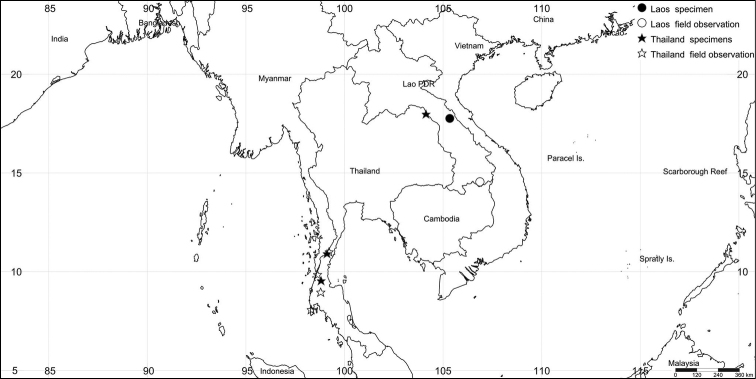
Geographic distribution of *D.dussaudii*. The solid star and star represent newly recorded localities in Thailand and the solid circle and circle represent localities in Laos. The map was created with SimpleMappr (http://www.simplemappr.net; Shorthouse 2010).

Laos. reçu le 4 October, 1913, *Dussaud 120* (lectotype designated here, P barcode P00721485; isolectotypes P barcode P00721486 and P02141495). Figs 1 and 3–5.

Tree, 8–15(–20) m tall, up to 20 cm in diameter; trunk straight, without buttresses; outer bark grey, smooth and sparsely lenticellate; inner bark thick, reddish-brown or blackish-brown; sapwood white. Young branches covered with hairs, persistent or later glabrous. Buds covered by bifarious scales, ca. 3.0 cm long, outside densely pubescent. Leaves alternate; petiole 8–9 mm long, with dense short brown hairs; blade subcoriaceous, oblong or elliptic-oblong, 12.0–16.2 × 3.8–5.0 cm, base attenuate to cuneate, apex acute to acuminate; mid-rib prominent and finely pubescent above, glabrous or slightly pubescent or puberulous below; secondary veins 7–10 on each side, grooved above, raised below; glabrous adaxially, glabrous to tomentose abaxially. Male inflorescences fasciculate, axillary or on older branches below the leaves, covered with bifarious scales at base. Male flowers 4-merous; pedicel ca. 1 mm long, pubescent; calyx tubular, with 4 short lobes, rounded at apex, 6.5–8.0 mm long, pubescent outside, glabrous inside; corolla white, salverform, pubescent outside, glabrous inside, tube ca. 8.0 mm long, cylindrical, lobes 4, lanceolate, 10.0 × 3.0–3.5 mm; stamens 12–16, arranged in 2 series, attached at base of corolla tube; filaments 1–3 mm long, sparsely pubescent; anthers triangular, ca. 1.8 mm long, apex apiculate, dehiscence sublateral. Female inflorescences 1-flowered, in the axils of leaves or on older branches below the leaves. Female flowers 4-merous; sessile or subsessile, pedicel up to ca. 3.0 mm long, bracteate; calyx green, tube ca. 6.0 mm long, pubescent outside, glabrous inside, lobes rounded, ca. 5.0 × 3.0 mm, pubescent outside, glabrous inside; corolla creamy-white to pale yellow, urceolate, tube ca. 9.0 mm long, ca. 7.0 mm in diameter, lobes elliptic, ca. 10.0 × 7.0 mm, pubescent outside, glabrous inside; staminodes 8, attached at the base of corolla tube; ovary globose, pubescent, 8-locular; style 1, ca. 1 mm long, glabrous, stigmas 4. Fruits globose or depressed-globose, covered with dense orange hairs, 3.8–5.0 × 4.0–5.0 cm, apex rounded, shortly apiculate, 8-locular; seeds 3–8 per fruit, light green when immature, turning yellow and orange when ripe; fruiting calyx divided to base, lobes ovate-oblong, spreading, ca. 4.0 × 5.0 mm, pubescent outside, glabrous inside; fruit stalk ca. 5.0 mm long; mesocarp 3.0–4.0 mm thick, cream with brown dots. Seeds ellipsoid to planoconvex, two faces flat and one face convex, glabrous, ca. 4.0 × 5.0 mm, black, endosperm smooth when young, but slightly ruminated when mature.

##### Additional specimens examined.

Laos. Khammouan: Nam Theun, Kaeng Luang, landing area near waterfall, 17°45'46"N, 105°20'21"E, 555 m alt., 7 November 2005, fr., *Newman et al. LAO 833* (**E**!, **L**!). Thailand. Nakhon Phanom Province: Ban Phaeng District, Phu Langka National Park, trail behind park headquarters, 17°59'06.60"N, 104°07'58.20"E, 197 m alt., 14 June 2013, fr., *Suddee et al. 4514* (**BKF**); Ban Phaeng District, Phai Lom, Phu Langka National Park, Tat Pho Waterfall, 26 December 2019, *Khammongkol 207* (**BKF**). Chumphon Province: Lamae District, 12 December 2014, *Sinbumroong 12122014-1* (fr.) (**BKF**); ibid., 8 May 2020, *Sinbumroong 08052020-1* (female fl.) (**BKF**); Tha Sae District, 10 December 2020, *Sinbumroong 10122020-1* (fr.) (**BKF**). Surat Thani Province: Tha Chana District, 4 April 2020, *Sinbumroong & Suekaew 04042020-1* (fr.) (**BKF**); Ban Ta Khun District, Ratchaprapa Dam, 12 Sep. 2020, *Sinbumroong 12092020-1* (male fl.) (**BKF**).

##### Distribution.

Laos, Thailand (Fig. 5).

##### Ecology.

Scattered along streams in tropical rain forests and dry evergreen forests. The species occurs in the understorey at altitudes of 100–300 m. The canopy of the tropical rainforest in Kaeng Krung National Park, Surat Thani, where the species occurs, is dominated by *Anisopteracostata* Korth., *Artocarpusrigidus* Blume, *Dipterocarpusgracilis* Blume, *Dipterocarpuskerrii* King, *Palaquiumimpressionervium* Ng and *Parashoreastellata* Kurz. *Dacryodesrostrata* (Blume) H.J. Lam forms a high sub-canopy with *Hopeaoblongifolia* Dyer, *Hydnocarpuscastaneus* Hook. f. & Thomson, *Mesuaferrea* L. and *Xerospermumnoronhianum* (Blume) Blume. Understorey species are *Barringtoniapauciflora* King, *Diospyrossumatrana* Miq., *Hydnocarpusnanus* King, *Koilodepaslongifolium* Hook. f. and *Microdesmiscaseariifolia* Planch. ex Hook.

##### Conservation status.

As the Laotian population remains unknown, we have classified the species as Data Deficient (DD) based on IUCN Red List Criteria (IUCN 2019).

##### Phenology.

Flowering May–October, fruiting May–April.

##### Note.

When mature, specimens from peninsular Thailand exhibit hairy branches, whereas those collected from Phu Langka National Park have glabrous or glabrescent branches. The Laos specimens match those from Phu Langka National Park.

As mentioned in Newman et al. (2007b), duplicates of Laotian specimens were deposited in three other herbaria in Laos, as well as at P. However, we were unable to study the specimens in Laos due to the COVID-19 pandemic. It is unsurprising that only a few specimens were collected from Laos because Laos has a low rate of botanical collection (Middleton et al. 2019).

##### GenBank accession no.

Sinbumroong 12122014-1: MZ457089 (*rbcL*), MZ457093 (*atpB*), MZ457101 (*matK* and *trnK* intron), MZ457097 (*ndhF*), MZ457105 (*trnL* intron and *trnL*-*trnF* spacer) and MZ457109 (*trnS*-*trnG* spacer). Sinbumroong & Suekaew 04042020-1: MZ457090 (*rbcL*), MZ457094 (*atpB*), MZ457102 (*matK* and *trnK* intron), MZ457098 (*ndhF*), MZ457106 (*trnL* intron and *trnL*-*trnF* spacer) and MZ457110 (*trnS*-*trnG* spacer). Sinbumroong 12092020-1: MZ457091 (*rbcL*), MZ457095 (*atpB*), MZ457103 (*matK* and *trnK* intron), MZ457099 (*ndhF*), MZ457107 (*trnL* intron and *trnL*-*trnF* spacer) and MZ457111 (*trnS*-*trnG* spacer). Khammongkol 207: MZ457092 (*rbcL*), MZ457096 (*atpB*), MZ457104 (*matK* and *trnK* intron), MZ457100 (*ndhF*), MZ457108 (*trnL* intron and *trnL*-*trnF* spacer) and MZ457112 (*trnS*-*trnG* spacer).

## Supplementary Material

XML Treatment for Diospyros dussaudii
